# 1-{2-[2-(1*H*-Benzimidazol-1-yl)eth­oxy]eth­yl}-1*H*-benzimidazol-3-ium hexa­fluoro­phosphate

**DOI:** 10.1107/S1600536810014534

**Published:** 2010-04-24

**Authors:** Sheng-Fu Sun, Jin Xu, Da-Bin Qin

**Affiliations:** a School of Chemistry and Chemical Engineering, China West Normal University, Nanchong 637002, People’s Republic of China

## Abstract

In the title salt, C_18_H_19_N_4_O^+^·PF_6_^−^, the dihedral angle between the benzimidazolium and benzimidazole ring systems is 16.24 (2)°. In the cation, a π–π inter­action is observed between the imidazolium ring and the benzene ring of the benzimidazole ring system [centroid–centroid distance = 3.5713 (11) Å]. The PF_6_^−^ ion is disordered over two sites, with occupancies of 0.895 (2) and 0.105 (2). In the crystal structure, pairs of N—H⋯N hydrogen bonds link the cations, forming centrosymmetric dimers. The dimers are linked *via* π–π inter­actions [centroid–centroid distance = 3.5606 (11) Å]. In addition, C—H⋯F hydrogen bonds are observed.

## Related literature

For the synthesis, see: Zeng *et al.* (2008 ▶). For general background to benzimidazole derivatives, see: Pal *et al.* (2007 ▶); Murru *et al.* (2009 ▶).
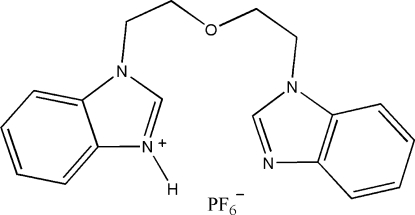


## Experimental

### 

#### Crystal data

C_18_H_19_N_4_O^+^·PF_6_^−^
*M* *_r_* = 452.34
Monoclinic, 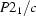

*a* = 10.5347 (18) Å
*b* = 13.771 (2) Å
*c* = 13.353 (2) Å
β = 92.507 (2)°
*V* = 1935.3 (6) Å^3^
*Z* = 4
Mo *K*α radiation
μ = 0.22 mm^−1^
*T* = 93 K
0.37 × 0.33 × 0.27 mm

#### Data collection

Rigaku SPIDER diffractometer
Absorption correction: multi-scan (*ABSCOR*; Higashi, 1995 ▶) *T*_min_ = 0.923, *T*_max_ = 0.943
11885 measured reflections
3959 independent reflections
3445 reflections with *I* > 2σ(*I*)
*R*_int_ = 0.026
Standard reflections: 0

#### Refinement

*R*[*F* ^2^ > 2σ(*F* ^2^)] = 0.040
*wR*(*F* ^2^) = 0.080
*S* = 1.00
3959 reflections
303 parameters
21 restraints
H atoms treated by a mixture of independent and constrained refinement
Δρ_max_ = 0.34 e Å^−3^
Δρ_min_ = −0.28 e Å^−3^

### 

Data collection: *RAPID-AUTO* (Rigaku/MSC, 2004 ▶); cell refinement: *RAPID-AUTO*; data reduction: *RAPID-AUTO*; program(s) used to solve structure: *SHELXS97* (Sheldrick, 2008 ▶); program(s) used to refine structure: *SHELXL97* (Sheldrick, 2008 ▶); molecular graphics: *XP* in *SHELXTL* (Sheldrick, 2008 ▶); software used to prepare material for publication: *SHELXL97*.

## Supplementary Material

Crystal structure: contains datablocks global, I. DOI: 10.1107/S1600536810014534/ci5075sup1.cif

Structure factors: contains datablocks I. DOI: 10.1107/S1600536810014534/ci5075Isup2.hkl

Additional supplementary materials:  crystallographic information; 3D view; checkCIF report

## Figures and Tables

**Table 1:** Hydrogen-bond geometry (Å, °)

*D*—H⋯*A*	*D*—H	H⋯*A*	*D*⋯*A*	*D*—H⋯*A*
N1—H1*N*⋯N4^i^	1.05 (2)	1.68 (2)	2.724 (2)	176 (2)
C4—H4⋯F3^ii^	0.95	2.41	3.130 (2)	133
C7—H7⋯F4^iii^	0.95	2.23	3.100 (2)	152
C9—H9*B*⋯F2^iii^	0.99	2.40	3.340 (2)	159
C11—H11*A*⋯F2^iv^	0.99	2.51	3.066 (2)	116
C16—H16⋯F4^v^	0.95	2.39	3.300 (2)	161
C18—H18⋯F6	0.95	2.38	3.298 (2)	163

Symmetry codes: (i) 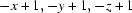; (ii) 
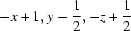
; (iii) 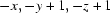; (iv) 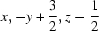; (v) 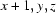.

**Fig. 1.**
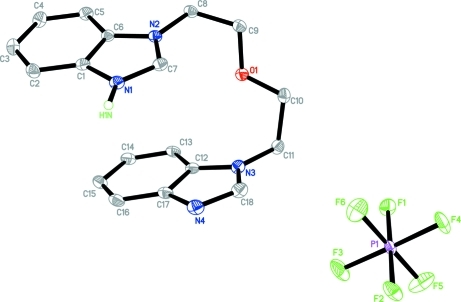
The asymmetric unit of the title compound. Displacement ellipsoids are drawn at the 30% probability level. C-bound H atoms have been omitted. For clarity, only the major disorder component is shown.

## supplementary crystallographic information

### Comment

Benzimidazole derivatives are an important class of heterocycles that are present in a number of biologically active compounds. These derivatives are also used as cyclic N-heterocyclic carbene (NHC) precursors (Murru *et al.*, 2009). A benzimidazole N-donor dinuclear palladacycle complex is used as an efficient Suzuki coupling catalyst (Pal *et al.*, 2007).

Bond lengths and angles in the ionic pairs (Fig. 1) are within normal ranges. The benzimidazolium and benzimidazole ring systems make a dihedral angle of 16.24 (2)°. In the cation, a π–π interaction is observed between N1/C1/C6/N2C7 and C12-C17 rings, with the ring centroids being separated by 3.5713 (11) Å.

In the crystal structure, pairs of N—H···N hydrogen bonds link cations to form centrosymmetric dimers. The dimers are linked via π–π interactions between the N1/C1/C6/N2C7 ring at (x, y, z) and the C12-C17 ring at (1-x, y-1/2, 1/2-z), with their centroids separated by 3.5606 (11) Å. In addition, C—H···F hydrogen bonds are observed (Table 1).

### Experimental

The title compound was prepared according to the reported procedure of Zeng *et al.*. (2008). Colourless single crystals suitable for X-ray diffraction were obtained by recrystallization from acetonitrile and ethyl ether.

### Refinement

The PF_6_^-^ ion is disordered over two sites with occupancies of 0.895 (2) and 0.105 (2). The U^ij^ values of atom pairs F1/F1', F2/F2', F3/F3, F4/F4', F5/F5' and F6/F6' were constrained to be equal. The corresponding distances in the two disorder components were restrained to be the same. Atom H1N was located in a difference map and refined freely. All other H atoms were placed in calculated positions [C–H = 0.95–0.99 Å] and refined in riding mode with U_iso_(H) = 1.2U_eq_(C).

### Crystal data

**Table:**

C_18_H_19_N_4_O^+^·PF_6_^−^	*F*(000) = 928
*M**_r_* = 452.34	*D*_x_ = 1.553 Mg m^−^^3^
Monoclinic, *P*2_1_/*c*	Mo *K*α radiation, λ = 0.71073 Å
Hall symbol: -P 2ybc	Cell parameters from 5714 reflections
*a* = 10.5347 (18) Å	θ = 3.1–27.5°
*b* = 13.771 (2) Å	µ = 0.22 mm^−^^1^
*c* = 13.353 (2) Å	*T* = 93 K
β = 92.507 (2)°	Block, colourless
*V* = 1935.3 (6) Å^3^	0.37 × 0.33 × 0.27 mm
*Z* = 4	

### Data collection

**Table:**

Rigaku SPIDER diffractometer	3959 independent reflections
Radiation source: Rotating Anode	3445 reflections with *I* > 2σ(*I*)
graphite	*R*_int_ = 0.026
ω scans	θ_max_ = 26.4°, θ_min_ = 3.1°
Absorption correction: multi-scan (*ABSCOR*; Higashi, 1995)	*h* = −13→10
*T*_min_ = 0.923, *T*_max_ = 0.943	*k* = −13→17
11885 measured reflections	*l* = −15→16

### Refinement

**Table:**

Refinement on *F*^2^	Primary atom site location: structure-invariant direct methods
Least-squares matrix: full	Secondary atom site location: difference Fourier map
*R*[*F*^2^ > 2σ(*F*^2^)] = 0.040	Hydrogen site location: inferred from neighbouring sites
*wR*(*F*^2^) = 0.080	H atoms treated by a mixture of independent and constrained refinement
*S* = 1.00	*w* = 1/[σ^2^(*F*_o_^2^) + (0.0259*P*)^2^ + 0.968*P*] where *P* = (*F*_o_^2^ + 2*F*_c_^2^)/3
3959 reflections	(Δ/σ)_max_ = 0.001
303 parameters	Δρ_max_ = 0.34 e Å^−^^3^
21 restraints	Δρ_min_ = −0.28 e Å^−^^3^

### Special details

**Table:**

Geometry. All esds (except the esd in the dihedral angle between two l.s. planes) are estimated using the full covariance matrix. The cell esds are taken into account individually in the estimation of esds in distances, angles and torsion angles; correlations between esds in cell parameters are only used when they are defined by crystal symmetry. An approximate (isotropic) treatment of cell esds is used for estimating esds involving l.s. planes.
Refinement. Refinement of *F*^2^ against ALL reflections. The weighted *R*-factor wR and goodness of fit *S* are based on *F*^2^, conventional *R*-factors *R* are based on *F*, with *F* set to zero for negative *F*^2^. The threshold expression of *F*^2^ > σ(*F*^2^) is used only for calculating *R*-factors(gt) etc. and is not relevant to the choice of reflections for refinement. *R*-factors based on *F*^2^ are statistically about twice as large as those based on *F*, and *R*- factors based on ALL data will be even larger.

### Fractional atomic coordinates and isotropic or equivalent isotropic displacement parameters (Å^2^)

**Table:**

	*x*	*y*	*z*	*U*_iso_*/*U*_eq_	Occ. (<1)
O1	0.20360 (11)	0.51931 (8)	0.18156 (8)	0.0247 (3)	
N1	0.53426 (13)	0.40531 (10)	0.31178 (10)	0.0243 (3)	
N2	0.41920 (13)	0.39824 (9)	0.17089 (10)	0.0218 (3)	
N3	0.26475 (13)	0.62988 (10)	0.35541 (10)	0.0253 (3)	
N4	0.39492 (14)	0.60700 (10)	0.48994 (10)	0.0294 (3)	
C1	0.61864 (16)	0.39760 (11)	0.23507 (12)	0.0229 (3)	
C2	0.75022 (16)	0.39314 (12)	0.23627 (13)	0.0285 (4)	
H2	0.8005	0.3964	0.2970	0.034*	
C3	0.80419 (17)	0.38372 (13)	0.14435 (14)	0.0319 (4)	
H3	0.8941	0.3801	0.1420	0.038*	
C4	0.73059 (17)	0.37927 (13)	0.05438 (13)	0.0311 (4)	
H4	0.7718	0.3730	−0.0072	0.037*	
C5	0.60018 (16)	0.38377 (12)	0.05330 (12)	0.0262 (4)	
H5	0.5499	0.3808	−0.0075	0.031*	
C6	0.54567 (15)	0.39293 (11)	0.14572 (12)	0.0218 (3)	
C7	0.41753 (16)	0.40521 (11)	0.27069 (12)	0.0245 (4)	
H7	0.3424	0.4095	0.3073	0.029*	
C8	0.31129 (15)	0.39532 (12)	0.09736 (12)	0.0242 (4)	
H8A	0.3030	0.3288	0.0698	0.029*	
H8B	0.3286	0.4397	0.0412	0.029*	
C9	0.18818 (16)	0.42403 (12)	0.14171 (13)	0.0262 (4)	
H9A	0.1185	0.4231	0.0895	0.031*	
H9B	0.1668	0.3780	0.1954	0.031*	
C10	0.10406 (16)	0.54786 (12)	0.24439 (12)	0.0272 (4)	
H10A	0.0890	0.4968	0.2947	0.033*	
H10B	0.0243	0.5584	0.2039	0.033*	
C11	0.14574 (16)	0.64079 (13)	0.29557 (12)	0.0277 (4)	
H11A	0.1568	0.6916	0.2442	0.033*	
H11B	0.0783	0.6627	0.3397	0.033*	
C12	0.38716 (16)	0.64002 (11)	0.32253 (12)	0.0231 (3)	
C13	0.43309 (16)	0.65920 (12)	0.22830 (12)	0.0262 (4)	
H13	0.3775	0.6687	0.1712	0.031*	
C14	0.56322 (17)	0.66369 (12)	0.22204 (13)	0.0295 (4)	
H14	0.5982	0.6764	0.1590	0.035*	
C15	0.64530 (18)	0.64993 (12)	0.30653 (14)	0.0314 (4)	
H15	0.7345	0.6539	0.2994	0.038*	
C16	0.59940 (17)	0.63079 (12)	0.39979 (13)	0.0294 (4)	
H16	0.6553	0.6214	0.4567	0.035*	
C17	0.46803 (17)	0.62576 (11)	0.40726 (12)	0.0249 (4)	
C18	0.27606 (18)	0.61067 (13)	0.45479 (12)	0.0299 (4)	
H18	0.2053	0.6008	0.4953	0.036*	
P1	−0.07196 (5)	0.71567 (4)	0.57610 (4)	0.02117 (17)	0.8952 (16)
F1	−0.10379 (15)	0.71525 (12)	0.45828 (8)	0.0350 (3)	0.8952 (16)
F2	−0.03912 (12)	0.71374 (11)	0.69440 (8)	0.0393 (4)	0.8952 (16)
F3	0.05729 (13)	0.77151 (12)	0.55925 (10)	0.0540 (4)	0.8952 (16)
F4	−0.19885 (12)	0.65494 (12)	0.59425 (9)	0.0479 (4)	0.8952 (16)
F5	−0.14535 (18)	0.81437 (11)	0.58739 (10)	0.0589 (5)	0.8952 (16)
F6	0.00255 (14)	0.61439 (10)	0.56578 (10)	0.0509 (4)	0.8952 (16)
P1'	−0.0834 (6)	0.7250 (5)	0.5725 (5)	0.141 (8)	0.1048 (16)
F1'	−0.0918 (11)	0.7157 (9)	0.4533 (4)	0.0350 (3)	0.1048 (16)
F2'	−0.0730 (10)	0.7334 (8)	0.6921 (5)	0.0393 (4)	0.1048 (16)
F3'	−0.0180 (10)	0.8283 (6)	0.5636 (8)	0.0540 (4)	0.1048 (16)
F4'	−0.1434 (9)	0.6186 (6)	0.5824 (8)	0.0479 (4)	0.1048 (16)
F5'	−0.2196 (8)	0.7721 (8)	0.5714 (8)	0.0589 (5)	0.1048 (16)
F6'	0.0549 (7)	0.6755 (8)	0.5745 (8)	0.0509 (4)	0.1048 (16)
H1N	0.560 (2)	0.4037 (15)	0.3887 (16)	0.052 (6)*	

### Atomic displacement parameters (Å^2^)

**Table:**

	*U*^11^	*U*^22^	*U*^33^	*U*^12^	*U*^13^	*U*^23^
O1	0.0252 (6)	0.0252 (6)	0.0238 (6)	−0.0009 (5)	0.0013 (5)	−0.0021 (5)
N1	0.0294 (8)	0.0246 (7)	0.0183 (7)	0.0024 (6)	−0.0043 (6)	−0.0007 (6)
N2	0.0255 (7)	0.0219 (7)	0.0177 (6)	0.0001 (6)	−0.0024 (5)	−0.0005 (5)
N3	0.0272 (8)	0.0310 (8)	0.0175 (7)	0.0061 (6)	−0.0033 (6)	−0.0007 (6)
N4	0.0360 (9)	0.0325 (8)	0.0192 (7)	0.0027 (6)	−0.0050 (6)	0.0010 (6)
C1	0.0294 (9)	0.0190 (8)	0.0200 (8)	0.0016 (6)	−0.0023 (7)	−0.0006 (6)
C2	0.0285 (9)	0.0297 (9)	0.0266 (9)	0.0011 (7)	−0.0083 (7)	0.0012 (7)
C3	0.0248 (9)	0.0350 (10)	0.0357 (10)	0.0030 (7)	0.0004 (8)	0.0041 (8)
C4	0.0323 (10)	0.0361 (10)	0.0252 (9)	0.0054 (8)	0.0048 (8)	0.0035 (8)
C5	0.0319 (10)	0.0281 (9)	0.0184 (8)	0.0021 (7)	−0.0026 (7)	0.0003 (7)
C6	0.0246 (9)	0.0180 (8)	0.0225 (8)	0.0011 (6)	−0.0026 (7)	−0.0001 (6)
C7	0.0301 (9)	0.0235 (8)	0.0198 (8)	0.0017 (7)	0.0007 (7)	−0.0002 (7)
C8	0.0261 (9)	0.0254 (9)	0.0204 (8)	−0.0013 (7)	−0.0059 (7)	−0.0011 (7)
C9	0.0259 (9)	0.0268 (9)	0.0255 (9)	−0.0024 (7)	−0.0030 (7)	−0.0015 (7)
C10	0.0230 (9)	0.0346 (10)	0.0240 (9)	0.0024 (7)	0.0012 (7)	0.0020 (7)
C11	0.0249 (9)	0.0357 (10)	0.0222 (8)	0.0076 (7)	−0.0027 (7)	−0.0003 (7)
C12	0.0281 (9)	0.0199 (8)	0.0207 (8)	0.0022 (7)	−0.0044 (7)	−0.0031 (6)
C13	0.0329 (10)	0.0262 (9)	0.0189 (8)	−0.0019 (7)	−0.0055 (7)	−0.0033 (7)
C14	0.0347 (10)	0.0285 (9)	0.0254 (9)	−0.0061 (7)	0.0015 (8)	−0.0051 (7)
C15	0.0288 (10)	0.0287 (9)	0.0362 (10)	−0.0045 (7)	−0.0039 (8)	−0.0048 (8)
C16	0.0332 (10)	0.0246 (9)	0.0294 (9)	−0.0008 (7)	−0.0120 (8)	−0.0027 (7)
C17	0.0325 (9)	0.0202 (8)	0.0212 (8)	0.0013 (7)	−0.0056 (7)	−0.0019 (6)
C18	0.0360 (10)	0.0346 (10)	0.0189 (8)	0.0048 (8)	−0.0004 (7)	0.0013 (7)
P1	0.0156 (3)	0.0302 (3)	0.0175 (4)	−0.0041 (2)	−0.0011 (2)	0.0053 (3)
F1	0.0370 (7)	0.0499 (7)	0.0182 (5)	0.0021 (5)	0.0012 (4)	0.0020 (5)
F2	0.0360 (9)	0.0587 (9)	0.0220 (5)	−0.0174 (6)	−0.0105 (5)	0.0105 (5)
F3	0.0427 (9)	0.0789 (11)	0.0405 (8)	−0.0350 (8)	0.0021 (6)	0.0096 (7)
F4	0.0266 (8)	0.0901 (11)	0.0269 (6)	−0.0273 (7)	−0.0006 (6)	0.0019 (7)
F5	0.0895 (13)	0.0551 (10)	0.0322 (8)	0.0370 (9)	0.0014 (8)	−0.0026 (7)
F6	0.0558 (9)	0.0451 (9)	0.0519 (8)	0.0174 (7)	0.0044 (7)	0.0118 (7)
P1'	0.167 (16)	0.22 (2)	0.030 (7)	−0.026 (15)	−0.032 (8)	0.006 (9)
F1'	0.0370 (7)	0.0499 (7)	0.0182 (5)	0.0021 (5)	0.0012 (4)	0.0020 (5)
F2'	0.0360 (9)	0.0587 (9)	0.0220 (5)	−0.0174 (6)	−0.0105 (5)	0.0105 (5)
F3'	0.0427 (9)	0.0789 (11)	0.0405 (8)	−0.0350 (8)	0.0021 (6)	0.0096 (7)
F4'	0.0266 (8)	0.0901 (11)	0.0269 (6)	−0.0273 (7)	−0.0006 (6)	0.0019 (7)
F5'	0.0895 (13)	0.0551 (10)	0.0322 (8)	0.0370 (9)	0.0014 (8)	−0.0026 (7)
F6'	0.0558 (9)	0.0451 (9)	0.0519 (8)	0.0174 (7)	0.0044 (7)	0.0118 (7)

### Geometric parameters (Å, °)

**Table:**

O1—C9	1.4224 (19)	C10—C11	1.507 (2)
O1—C10	1.4266 (19)	C10—H10A	0.99
N1—C7	1.325 (2)	C10—H10B	0.99
N1—C1	1.389 (2)	C11—H11A	0.99
N1—H1N	1.05 (2)	C11—H11B	0.99
N2—C7	1.337 (2)	C12—C13	1.393 (2)
N2—C6	1.390 (2)	C12—C17	1.400 (2)
N2—C8	1.470 (2)	C13—C14	1.378 (2)
N3—C18	1.353 (2)	C13—H13	0.95
N3—C12	1.387 (2)	C14—C15	1.404 (2)
N3—C11	1.465 (2)	C14—H14	0.95
N4—C18	1.319 (2)	C15—C16	1.381 (3)
N4—C17	1.398 (2)	C15—H15	0.95
C1—C2	1.387 (2)	C16—C17	1.394 (2)
C1—C6	1.392 (2)	C16—H16	0.95
C2—C3	1.381 (2)	C18—H18	0.95
C2—H2	0.95	P1—F5	1.5742 (14)
C3—C4	1.402 (3)	P1—F3	1.5883 (13)
C3—H3	0.95	P1—F1	1.5942 (12)
C4—C5	1.375 (2)	P1—F2	1.6025 (12)
C4—H4	0.95	P1—F4	1.6041 (12)
C5—C6	1.389 (2)	P1—F6	1.6092 (14)
C5—H5	0.95	P1'—F5'	1.574 (3)
C7—H7	0.95	P1'—F3'	1.588 (3)
C8—C9	1.502 (2)	P1'—F1'	1.595 (3)
C8—H8A	0.99	P1'—F2'	1.601 (3)
C8—H8B	0.99	P1'—F4'	1.603 (3)
C9—H9A	0.99	P1'—F6'	1.608 (3)
C9—H9B	0.99		
			
C9—O1—C10	113.54 (12)	C10—C11—H11B	109.1
C7—N1—C1	107.87 (14)	H11A—C11—H11B	107.8
C7—N1—H1N	126.9 (11)	N3—C12—C13	132.02 (15)
C1—N1—H1N	125.0 (11)	N3—C12—C17	105.74 (14)
C7—N2—C6	107.41 (13)	C13—C12—C17	122.24 (16)
C7—N2—C8	128.63 (14)	C14—C13—C12	116.68 (16)
C6—N2—C8	123.95 (13)	C14—C13—H13	121.7
C18—N3—C12	106.67 (14)	C12—C13—H13	121.7
C18—N3—C11	126.26 (15)	C13—C14—C15	121.63 (16)
C12—N3—C11	127.04 (13)	C13—C14—H14	119.2
C18—N4—C17	105.03 (14)	C15—C14—H14	119.2
C2—C1—N1	131.78 (15)	C16—C15—C14	121.52 (17)
C2—C1—C6	121.47 (15)	C16—C15—H15	119.2
N1—C1—C6	106.75 (14)	C14—C15—H15	119.2
C3—C2—C1	116.36 (16)	C15—C16—C17	117.52 (16)
C3—C2—H2	121.8	C15—C16—H16	121.2
C1—C2—H2	121.8	C17—C16—H16	121.2
C2—C3—C4	122.12 (17)	C16—C17—N4	130.48 (16)
C2—C3—H3	118.9	C16—C17—C12	120.41 (16)
C4—C3—H3	118.9	N4—C17—C12	109.11 (15)
C5—C4—C3	121.46 (16)	N4—C18—N3	113.45 (16)
C5—C4—H4	119.3	N4—C18—H18	123.3
C3—C4—H4	119.3	N3—C18—H18	123.3
C4—C5—C6	116.52 (16)	F5—P1—F3	91.33 (10)
C4—C5—H5	121.7	F5—P1—F1	90.84 (8)
C6—C5—H5	121.7	F3—P1—F1	90.37 (7)
C5—C6—N2	131.09 (15)	F5—P1—F2	90.33 (7)
C5—C6—C1	122.08 (15)	F3—P1—F2	89.90 (7)
N2—C6—C1	106.83 (14)	F1—P1—F2	178.79 (8)
N1—C7—N2	111.14 (15)	F5—P1—F4	91.13 (10)
N1—C7—H7	124.4	F3—P1—F4	177.42 (10)
N2—C7—H7	124.4	F1—P1—F4	90.37 (7)
N2—C8—C9	112.67 (13)	F2—P1—F4	89.30 (6)
N2—C8—H8A	109.1	F5—P1—F6	179.35 (8)
C9—C8—H8A	109.1	F3—P1—F6	89.03 (8)
N2—C8—H8B	109.1	F1—P1—F6	89.69 (8)
C9—C8—H8B	109.1	F2—P1—F6	89.14 (8)
H8A—C8—H8B	107.8	F4—P1—F6	88.51 (9)
O1—C9—C8	107.68 (13)	F5'—P1'—F3'	91.6 (3)
O1—C9—H9A	110.2	F5'—P1'—F1'	90.8 (3)
C8—C9—H9A	110.2	F3'—P1'—F1'	90.2 (3)
O1—C9—H9B	110.2	F5'—P1'—F2'	90.1 (3)
C8—C9—H9B	110.2	F3'—P1'—F2'	89.9 (3)
H9A—C9—H9B	108.5	F1'—P1'—F2'	179.1 (4)
O1—C10—C11	107.08 (13)	F5'—P1'—F4'	90.8 (3)
O1—C10—H10A	110.3	F3'—P1'—F4'	177.5 (4)
C11—C10—H10A	110.3	F1'—P1'—F4'	90.2 (3)
O1—C10—H10B	110.3	F2'—P1'—F4'	89.6 (3)
C11—C10—H10B	110.3	F5'—P1'—F6'	179.1 (4)
H10A—C10—H10B	108.6	F3'—P1'—F6'	89.2 (3)
N3—C11—C10	112.69 (14)	F1'—P1'—F6'	89.7 (3)
N3—C11—H11A	109.1	F2'—P1'—F6'	89.5 (3)
C10—C11—H11A	109.1	F4'—P1'—F6'	88.4 (3)
N3—C11—H11B	109.1		
			
C7—N1—C1—C2	−179.03 (17)	C9—O1—C10—C11	169.54 (13)
C7—N1—C1—C6	0.03 (17)	C18—N3—C11—C10	−94.78 (19)
N1—C1—C2—C3	178.85 (16)	C12—N3—C11—C10	87.35 (19)
C6—C1—C2—C3	−0.1 (2)	O1—C10—C11—N3	−58.98 (17)
C1—C2—C3—C4	0.3 (3)	C18—N3—C12—C13	179.45 (17)
C2—C3—C4—C5	−0.2 (3)	C11—N3—C12—C13	−2.3 (3)
C3—C4—C5—C6	0.0 (2)	C18—N3—C12—C17	0.01 (17)
C4—C5—C6—N2	−179.17 (16)	C11—N3—C12—C17	178.22 (15)
C4—C5—C6—C1	0.2 (2)	N3—C12—C13—C14	−179.43 (16)
C7—N2—C6—C5	179.09 (16)	C17—C12—C13—C14	−0.1 (2)
C8—N2—C6—C5	−0.4 (3)	C12—C13—C14—C15	−0.2 (2)
C7—N2—C6—C1	−0.31 (17)	C13—C14—C15—C16	0.3 (3)
C8—N2—C6—C1	−179.76 (13)	C14—C15—C16—C17	−0.1 (2)
C2—C1—C6—C5	−0.1 (2)	C15—C16—C17—N4	179.34 (16)
N1—C1—C6—C5	−179.29 (14)	C15—C16—C17—C12	−0.2 (2)
C2—C1—C6—N2	179.35 (14)	C18—N4—C17—C16	−179.82 (17)
N1—C1—C6—N2	0.17 (17)	C18—N4—C17—C12	−0.29 (18)
C1—N1—C7—N2	−0.23 (18)	N3—C12—C17—C16	179.76 (14)
C6—N2—C7—N1	0.34 (18)	C13—C12—C17—C16	0.3 (2)
C8—N2—C7—N1	179.76 (14)	N3—C12—C17—N4	0.17 (17)
C7—N2—C8—C9	13.2 (2)	C13—C12—C17—N4	−179.34 (14)
C6—N2—C8—C9	−167.43 (14)	C17—N4—C18—N3	0.31 (19)
C10—O1—C9—C8	−167.47 (13)	C12—N3—C18—N4	−0.21 (19)
N2—C8—C9—O1	58.10 (17)	C11—N3—C18—N4	−178.43 (15)

### Hydrogen-bond geometry (Å, °)

**Table:**

*D*—H···*A*	*D*—H	H···*A*	*D*···*A*	*D*—H···*A*
N1—H1N···N4^i^	1.05 (2)	1.68 (2)	2.724 (2)	176 (2)
C4—H4···F3^ii^	0.95	2.41	3.130 (2)	133
C7—H7···F4^iii^	0.95	2.23	3.100 (2)	152
C9—H9B···F2^iii^	0.99	2.40	3.340 (2)	159
C11—H11A···F2^iv^	0.99	2.51	3.066 (2)	116
C16—H16···F4^v^	0.95	2.39	3.300 (2)	161
C18—H18···F6	0.95	2.38	3.298 (2)	163

Symmetry codes: (i) −*x*+1, −*y*+1, −*z*+1; (ii) −*x*+1, *y*−1/2, −*z*+1/2; (iii) −*x*, −*y*+1, −*z*+1; (iv) *x*, −*y*+3/2, *z*−1/2; (v) *x*+1, *y*, *z*.
